# A tool to measure the attributes of receiving IV therapy in a home versus hospital setting: the Multiple Sclerosis Relapse Management Scale (MSRMS)

**DOI:** 10.1186/1477-7525-9-80

**Published:** 2011-09-26

**Authors:** Afsane Riazi, Bernadette Porter, Jeremy Chataway, Alan J Thompson, Jeremy C Hobart

**Affiliations:** 1 Department of Psychology, Royal Holloway, University of London, Surrey, TW20 0EX, UK; 2 Neurological Outcome Measures Unit, Institute of Neurology, Queen Square, London, WC1N 3BG, UK; 3 National Hospital for Neurology and Neurosurgery, Queen Square, London, WC1N 3BG, UK; 4 Peninsula Medical School, Derriford Hospital, Plymouth, Devon, PL6 8BX, UK

## Abstract

**Background:**

Intravenous steroids are routinely used to treat disabling relapses in multiple sclerosis (MS). Theoretically, the infusion could take place at home, rather than in hospital. Findings from other patient populations suggest that patients may find the experiences of home relapse management more desirable. However, formal comparison of these two settings, from the patients' point of view, was prevented by the lack of a clinical scale. We report the development of a rating scale to measure patient's experiences of relapse management that allowed this question to be answered confidently.

**Methods:**

Scale development had three stages. First, in-depth interviews of 21 MS patients generated a conceptual model and pool of potential scale items. Second, these items were administered to 160 people with relapsing-remitting MS. Standard psychometric techniques were used to develop a scale. Third, the psychometric properties of the scale were evaluated in a randomised controlled trial of 138 patients whose relapses were managed either at home or hospital.

**Results:**

A preliminary conceptual model with eight dimensions, and a pool of 154 items was generated. From this we developed the MS Relapse Management Scale (MSRMS), a 42-item with four subscales: access to care (6 items), coordination of care (11 items), information (7 items), interpersonal care (18 items). The MSRMS subscales satisfied most psychometric criteria but had notable floor effects.

**Conclusions:**

The MSRMS is a reliable and valid measure of patients' experiences of MS relapse management. The high floor effects suggest most respondents had positive care experiences. Results demonstrate that patients' experiences of relapse management can be measured, and that the MSRMS is a powerful tool for determining which services to develop, support and ultimately commission.

## Background

Most people with multiple sclerosis (MS) follow an initial course of relapsing-remitting MS (RRMS), although many people go on to the phase of secondary progressive MS (SPMS), in which disability accumulates steadily with or without superimposed relapses [1]. It has been estimated that 8-10, 000 relapses will occur in the UK per annum [2], with only 60% recovering completely [3]. The standard treatment of acute disabling relapses is steroid based, often intravenous methylprednisolone (IVMP), administered at hospital or, rarely, in the home setting [4].

In other disciplines, such as cancer chemotherapy, it is acceptable practice to administer IV therapies at home, with clear benefits being demonstrated in compliance, satisfaction and cost [5-7]. It is therefore important to extend this model to the use of IVMP for disabling MS relapses, so that a clear policy decision can be made: hospital or community? Whilst it is likely that home administration of IVMP may be more suitable from the patients' perspective, a randomised controlled trial (RCT) is required to compare the health care experiences in these settings. Yet, to do this an explicit and valid outcome measure to quantify patients' experiences of relapse management is needed as the primary outcome. Although patient "satisfaction" ratings are often used as outcome measures when evaluating medical care from the patients' perspective, they have limitations. Typically, they result in very positive ratings that do not discriminate between people, and are insensitive to specific problems in care delivery [8]. This is probably because "satisfaction" is an ill-defined term. It is more useful clinically, to refine the measurement of patients' experiences by quantifying the main aspects of relapse management that people with MS find important. Ratings on these variables can then be compared across different treatments. This article reports the development of a rating scale measuring the essential aspects of patients' experiences in MS relapse management, to be used in a trial of delivery of IV steroids.

## Methods

### Overview

The scale was developed in three stages. First, in-depth interviews of 21 MS patients generated a conceptual model of relapse management and pool of potential scale items. Second, this pool of items was administered by postal survey to 160 people with relapsing-remitting MS, and standard psychometric techniques were used to develop a rating scale. Third, the scale was evaluated for five psychometric properties in a randomised controlled trial of 138 patients whose relapses were managed at either home or hospital.

#### Stage 1: Conceptual model formation and item generation

In-depth, one-to-one, interviews focusing specifically on people's experiences of relapse management were conducted with MS patients recently treated for relapses with IVMP. Patients were recruited from two UK clinical sites: the National Hospital for Neurology and Neurosurgery in London (NHNN), and Derriford Hospital in Plymouth. The ethical committees of both hospitals approved the study.

English-speaking adults, 18 years and over, were eligible for interview. To capture a full range of patient experiences, patients were chosen to ensure a wide variance of age, sex, disease duration and place of relapse management (home, outpatient, inpatient). Interviews were taped recorded, transcribed and content analysed using WinMax qualitative software [9]. In addition, a comprehensive literature review identified relevant areas and potential items for inclusion.

This qualitative stage had two goals. First, to identify the main components of relapse management that patients define as important. Second, to generate a pool of items that would eventually attempt to manifest each of these components as a rating scale. In practice, this was an iterative process in which different ideas about the key components, and how the items might be grouped were considered and integrated. This process continued until items were grouped under the most clinically appropriate component and a preliminary questionnaire was produced. This questionnaire was pre-tested in a small group of MS patients (recently treated for relapses) to identify and clarify ambiguities in wording and meaning.

#### Stage 2: Item reduction and scale development

The questionnaire developed in stage 1 was posted to 379 people with relapsing-remitting MS on a disease modifying drugs database attending NHNN. The booklet also contained two disease specific measures of MS impact: the Multiple Sclerosis Impact Scale (MSIS-29; [10]) and the Multiple Sclerosis Walking Scale (MSWS-12; [11]). To encourage high response rates we used personalised letters, standardised instructions, and two reminders for non-responders at three and five weeks. Data collection was closed three weeks after posting the second reminder

Traditional psychometric methods ([12]; [13]) were used to construct the final scale from the preliminary questionnaire. Essentially, we examined the item groups for each of the main component of relapse management defined by the conceptual model. For each group the items with the better measurement properties, that formed a clinically and statistically cohesive set, were retained to form the final subscales. The full psychometric criteria are presented in Table 1[14-19].

**Table 1:** Summary of psychometric properties evaluated and the criteria used for determining the adequacy of the MSRMS

	Criterion for adequacy
**Item analysis and scale development**	Items were eliminated due to:
	high missing data (> 10%); maximum endorsement frequencies (percentage of responses for the most frequently endorsed category) > 80%; sum of endorsement frequencies for any 2 adjacent item response categories < 10%; For each item that correlated highly with another item (> = 0.75), the item with the least favourable psychometric properties (on the other criteria above) was eliminated
	In principal components factor analysis (a method for reducing data while retaining those characteristics of the dataset that contribute most to its variance):
	Items that did not correlate (< 0.30) with the first unrotated component were removed, as it indicates that the items are not measuring a single underlying construct Items that did not load on any factors (< 0.40) or cross-loaded on two or more factors (< 0.20) were removed (the aim was to achieve a *simple structure*, that is, each item should be associated with one and only one factor)
	
**Data quality**	Missing item data < 10%
	High % computable scale scores
	
**Scaling assumptions**	Similar response-option frequency distributions
	Similar mean scores and variances
	Similar and substantial (*r *> 0.30) item-total correlations [14]
	
**Targeting**	Scores span the full scale range [15]
	Mean scores near the midpoint [16]
	Floor and ceiling effects (endorsement at the bottom and the highest end of the scale) < 20% [17]
	
**Reliability**	Cronbach's alpha (an indicator of internal consistency, or the extent to which items are interrelated) > 0.70 [18]
	
**Validity**	Discriminant construct validity, (evidence that the scale do not correlate with dissimilar measures) evidenced by low correlations (r < 0.30) between all subscales of the MSRMS and the Multiple Sclerosis Impact Scale (MSIS-29) [9] scales and the Medical Outcomes Study Short-form Health Survey SF-36 scales (SF-36) [19] Group differences validity, (the ability of the MSRMS scores to differentiate groups expected to differ) evidenced by stepwise mean differences in MSRMS scores for groups defined by patients' response to the single item question: *"Overall, how would you rate the quality of care you received for your relapse? 1 = poor, 2 = fair, 3 = good, 4 = very good, 5 = excellent"*

We also performed the following preliminary tests on each subscale of the instrument (Multiple Sclerosis Relapse Management Scale: MSRMS): data quality (missing data for each item), scaling assumptions (evidence that items in the same subscale can be summed to produce scale scores), targeting of scale to the sample (score distributions), reliability (the extent to which the scales are free from random error), and validity (evidence that the scales are measuring what they intend to measure) (Table 1). These psychometric properties are fully described in previous publications [10,13].

#### Stage 3: Psychometric evaluation of the MSRMS

One hundred and thirty-eight people were enrolled in a RCT of home versus outpatient management of MS relapses (for more information regarding the two groups in the trial, adverse events, and outcomes of the trial, please see [20]). Each person completed the MSRMS 1 week after receiving their course of treatment (methylprednisolone, 1 g/day, for three days). At the same time patients were asked to answer a single global question, *"Overall, how would you rate the quality of care you received for your relapse? 1 = poor, 2 = fair, 3 = good, 4 = very good, 5 = excellent"*. The SF-36 [19], a generic health status measure, the MSIS-29 [10], the MSWS-12 [11], and Kurtzke's Expanded Disability Status Scale (EDSS) [21], were also completed on the first day of treatment and 6 weeks follow-up. These scales were used as validating instruments, and to compare the samples in the two arms pre and post treatment.

### Analyses

We re-examined the same five psychometric properties of the MSRMS (data quality, scaling assumptions, targeting, reliability, and validity) in data from the RCT. In addition, we undertook more in-depth examinations of validity. First, we examined convergent and discriminate validity by correlating MSRMS scores with scores from the SF-36, MSIS-29, MSWS-12, and EDSS. Second, we examined group differences validity by comparing the mean scores of the MSRMS subscales in groups defined by patients' responses to the global question (*"Overall, how would you rate the quality of care you received for your relapse?*)*"*. Finally, we compared the psychometric properties of the MSRMS in the two independent samples (Stage 2 and Stage 3) to enable us to comment on the stability of the instrument.

## Results

### Stage 1: Conceptual model formation and item generation

Twenty-one people with MS were interviewed (Table 2), and their transcripts content analysed. This generated conceptual ideas about the main areas of relapse management, with around 1000 statements on people's experiences. Statements were grouped into clinically meaningful themes, and examined for redundancy. At this stage we were over inclusive in developing items from patients' statements. This qualitative work generated a preliminary conceptual model of patients' experiences of relapse management, and a preliminary 154-item questionnaire. Eight clinically relevant areas emerged: *access to care, coordination of care, physical comfort, technical aspects of care, involvement of family and friends, interpersonal care, attitude of health care professionals *and *information*. Each item was given individualised response options appropriate for that item. This is consistent with the Picker surveys [22] that also focus on measuring patients' experiences of care rather than satisfaction per se. Pre-testing of the questionnaire in 16 patients at NHNN undergoing relapse management resulted in minimal changes in wording.

**Table 2:** Characteristics of the three samples

	Interview	Postal survey	RCT
**N**	21	160	132
	%	%	%
**Gender**			
Female	52	70	76
**Age**			
Mean (sd)	39 (10)	40 (9)	39 (9)
Range	24-56	21-70	18-66
**Years since diagnosis**			
Mean (sd)	6 (6)	9 (7)	8 (7)
Range	0-21	0-39	0-33
**Ethnicity**			
White	86	81	77
**Mobility indoors**			
Walk unaided	57	55	62
**Employment status**			
Employed	61	42	50
**Location treated**			
Outpatients	48	29	49
Inpatients	19	36	0
Home	33	2	51
**Relapse severity**^**1**^			
Mild	N/A	20	10
Moderate	N/A	44	57
Severe	N/A	36	34
**Education**			
Degree or equivalent	N/A	43	60
**Marital Status**			
Married	N/A	46	50

N/A = not collected

### Stage 2: Item reduction and development of scales

A total of 379 questionnaires were posted and 296 people responded. Of these, 136 people responded by saying they had not had a relapse in the last 2 years. Thus 243 people (379 minus 136) were eligible to participate. 160 people returned completed questionnaires, which equals to 66% (160/243) response rate (Table 2).

#### Psychometric analyses

##### Item analysis and scale development

A total of 112 items from the original item pool was deleted. The reasons for deletion included: items did not meet the initial psychometric criteria (51 items), items did not load on the first unrotated factor (18 items), items did not load or cross-load on two or more factors (43 items) (Table 1). The remaining 42 items were grouped into four subscales rather than the eight domains of the preliminary conceptual model. One domain (*involvement of family and friends*) was removed because its items were not relevant for people who were isolated, or whose family and friends were not closely involved in their care experiences. Two domains (*attitude of health care professionals; interpersonal care*) were combined to form a single domain (*interpersonal care*) because they formed a more clinical and statistical cohesive set together rather than existing as separate scales. For the same reasons, three domains (*physical comfort, technical aspects of care, coordination of care*) were combined to form a single domain (*coordination of care*). This iterative process resulted in a 42-item scale (MS Relapse Management Scale, MSRMS), which measures four aspects of patients' experiences of relapse management: Access to Care (6 items), Coordination of Care (11 items), Information (7 items), and Interpersonal Care (18 items) (see Additional File 1 for Copy of the MSRMS and see Additional File 2 for Scoring methods for the MSRMS).

##### Preliminary psychometric analyses of MSRMS: postal survey sample

For all four MSRMS subscales, the vast majority of psychometric tests were satisfied supporting their reliability and validity. Full details are available from the authors. However, in brief, data quality was high (there were few missing data) implying that the scales can be completed successfully, and that the items are relevant, and not confusing or upsetting to people. Tests of scaling assumptions were satisfied, implying that the items in each subscale can be summed to produce a single subscale score. Subscale scores were well targeted to the sample indicating that this was an adequate sample of people with MS in which to examine the measurement properties of the MSRMS. All indicators of reliability exceeded recommended criteria, indicating that the total scores for each subscale were reliable estimates. The pattern and magnitude of correlations between the four MSRMS subscales and other rating scales (MSIS-29, MSWS-12, SF-36, EDSS), and MSRMS correlations with age and disease duration were consistent with a priori hypothesis. These findings provide evidence to support the validity of the MSRMS as they imply that the four MSRMS subscales were measuring related but distinct constructs, were unrelated to age and disease duration, and were measuring concepts distinct from those measured by the other rating scales.

### Stage 3: Psychometric evaluation of the MSRMS: RCT sample

Six patients had missing data at 6 weeks follow-up and were excluded from analysis. The characteristics of the remaining 132 people are shown in Table 2 and the psychometrics of the MSRMS in Tables 3, 4, and 5.

**Table 3:** Data quality, scaling assumptions, acceptability, and reliability of MSRMS

Psychometric Property	*Access to care* *(6 items)*	*Information* *(7 items)*	*Coordination of care* *(11 items)*	*Interpersonal care* *(18 items)*
**Data quality**				
Item missing data %	0-2.3	0-1.5	0-3.8	0
Computable scale scores %	100	99	100	100
**Scaling assumptions**				
Item mean scores	1.2-1.8	1.1-2.5	1.1-1.7	1.1-1.8
Item SD	0.59-0.91	0.41-1.0	0.22-0.88	0.30-1.11
Item-total correlation	0.42-0.70	0.21-0.79	0.00-0.57	0.26-0.63
**Targeting**				
Scale range	0-100	0-100	0-100	0-100
Score range	0-88.9	0-83.3	0-39.4	0-51.0
Mean score (SD)	17.9 (19.7)	30.6 (21.2)	9.9 (9.8)	9.2 (10.1)
Floor/ceiling effect %	21.2/0	10.8/0	20.5/0	28.0/0
**Reliability**				
Cronbach's alpha	0.80	0.87	0.73	0.84
SEM^2^	3.94	2.76	2.65	1.61

**Table 4:** MSRMS: intercorrelations^3 ^and correlations with other variables and scales

	*Access to care*	*Information*	*Co-ordination of care*	*Interpersonal Care*
***Access to Care***	1.0	0.42	0.36	0.33
***Information***	-	-	0.39	0.49
***Co-ordination of care***	-	-	-	0.46
				
***Age***	-0.12	0.13	-0.03	-0.09
***quality of care***^**4**^	-0.28	-0.31	-0.49	-0.38
				
***MSIS-29 physical***	0.19	0.05	0.05	-0.02
***MSIS-29*** ***Psychological***	0.20	0.09	0.10	0.06
***MS Walking***	0.11	0.09	-0.02	-0.03
				
***SF-36 Role Emotional***	-0.18	-0.12	-0.06	-0.11
***SF-36 Role Physical***	-0.10	-0.17	-0.07	-0.01
***SF-36 Pain***	-0.19	-0.07	0.03	-0.01
***SF-36 Vitality***	-0.17	-0.26	-0.03	-0.09
***SF-36 GHP***	-0.18	-0.18	-0.07	-0.00
***SF-36 Social***	-0.18	-0.16	-0.13	-0.05
***SF-36 Physical***	-0.07	-0.05	0.02	0.06
***SF-36 Mental***	-0.21	-0.16	-0.08	-0.10
***EDSS***	0.10	0.15	0.01	0.03

**Table 5:** Group differences validity analyses

**Groups determined by "quality of care" question**^**5**^	MSRMS subscales: mean (sd)			
	**Access to care**	**Coordination of care**	**Information**	**Interpersonal Care**

**'Good' or 'Very good' (N = 29)**	24.7 (17.3)	19.5 (10.8)	43.7 (21.0)	17.3 (12.9)
**'Excellent' (N = 98)**	15.7 (20.0)	6.8 (7.3)	26.5 (19.6)	6.9 (7.9)
***t (p)***^**6**^	2.2 (0.030)	7.3 (< 0.001)	4.1 (< 0.001)	5.3 (< 0.001)

#### Data quality (Table 3)

Item level missing data for all scales was low (max = 3.8%). Total scores could be computed for at least 99% of the sample, suggesting that the scales can be successfully completed by patients.

#### Scaling assumptions (Table 3)

For all scales, frequency distributions for items were quite symmetrical; items within each scale had similar mean scores and standard deviations implying that they contribute equally to the variance of the total score, and can be summed without weighting. Item-total scale correlations for all scales were satisfactory implying that the items in each scale contain a similar proportion of information concerning the construct being measured, except for one item in the 11-item Coordination of Care scale. These findings support the summing of items to generate subscale score.

#### Targeting (Table 3)

For all subscales, the scores did not span the entire scale range. None of the patients scored the highest possible score (indicating worse experience) for each of the subscales. All scales had notable floor effects (range 10.8% to 28%) but no ceiling effects. These results imply that most patients reported positive care experiences.

#### Reliability (Table 3)

All internal consistency estimates exceeded the recommended criteria (> 0.70). This indicates that the total scores for each subscale are reliable estimates. For each scale, the standard error of measurement, an indication of the amount of variation or spread in the measurement error, is relatively low.

#### Validity (Table 4)

The direction, magnitude and pattern of correlations among the four MSRMS subscales, and between the MSRMS subscales and other scales, were consistent with predictions. This supported the validity (convergent and discriminate construct) of the MSRMS.

More specifically, correlations among the MSRMS subscales (0.33-0.49) were moderate; suggesting they are measuring related but different constructs. Correlations between the four MSRMS subscales and the global quality of care question were low-to-moderate (0.28 - 0.49), suggesting that they are measuring related but different constructs.

As predicted, correlations among the four different aspects of patients experiences (i.e. MSRMS subscales), and correlations between these four experiences and the global indicator of quality of care (i.e. MSRMS with quality of care question), were higher than correlations of the MSRMS subscales with the MSIS-29, MSWS-12, SF-36, EDSS, and age.

Similarly, correlations between the MSRMS subscales and the MS-specific scales (MSIS-29 physical and psychological scales; MS Walking scale) were low (< 0.30), indicating that they are measuring different constructs. Low correlations were also found between the MSRMS subscales and the SF-36 scale, and between the MSRMS subscales and the EDSS.

Further evidence for validity (group differences validity) was provided by examining MSRMS scores for groups of people defined by their answer to the question *"Overall, how would you rate the quality of care you received for your relapse? 1 = poor, 2 = fair, 3 = good, 4 = very good, 5 = excellent"*. As only five people answered "poor" or "fair" we compared mean MSRMS scores for those reported whose care was good/very good with those who reported it to be excellent. Table 5 shows that the MSRMS subscales demonstrated a decrease in mean scores (indicating better experiences, significantly so for three subscales) associated with better evaluation of care as indicated by the 5-point scale.

#### Stability of MSRMS across samples

These results, from an independent sample of people, supported those from the preliminary psychometric analysis of the MSRMS providing further evidence to supporting its psychometric adequacy. However, there were notable differences in score distributions (range, means, floor effects) across the two samples (postal survey and RCT). The postal survey sample had better score distributions that met the psychometric criteria. The score distributions of the RCT sample were more skewed towards the better end of the scale, with patients reporting better experiences of care.

## Discussion

We have described the development of an outcome measure for quantifying patients' experiences of treatment for MS relapses, to be used as the primary outcome measure in a randomised controlled trial of service delivery [20]. Importantly, the MSRMS satisfies current health service aspirations towards a patient led service [23]. It is a patient reported measure, which enables relapse management to be evaluated from their perspective. In addition, patients themselves determined the domains of the MSRMS, therefore allowing them to define how services are evaluated.

The MSRMS satisfied most published criteria for robust measurement, with its psychometric performance being reasonably constant across two independent samples. However, scores in the postal survey sample were better distributed than in the RCT sample. There could be a number of reasons for this, including differences in the characteristics of the sample. The RCT sample included more people who received steroids at home, who may have reported better experiences of relapse management [20], due to a number of reasons, for example, the RCT sample may have known that outcomes were being assessed, whereas the postal survey sample received standard NHS care, which may have been more variable.

All four MRMS subscales had notable floor effects in the RCT sample indicating that the minimum possible score was commonly seen. From a clinical perspective, this finding might be taken to imply that many people considered that their relapse management could not have been better. This could, of course, be due to a number of reasons, for example, patients may not have wished to be critical about a service on which they rely, or they may have a positive rapport with the care team that make them forgive any deficiencies. Also from a conceptual perspective, it seems unlikely that care for these people could not have improved, even though floor effects and skewed positive responses are common in surveys that elicit patients' views of their health care [24]. Alternatively the floor effects could represent limitations in the measurement range covered by our scale. A qualitative evaluation of people at the floor end of the scale should help to determine whether their experiences could be improved, and will provide an evidence base for future development of the MSRMS.

The fact that we removed almost 70% of items from the preliminary scale may suggest that important information regarding patients' views on relapse management may have been discarded. However, the aim of the item generation stage was to be as inclusive of a wide range of patients' views as possible (inpatient, outpatient and home). Our over-inclusiveness at the item generation stage led to the inclusion of items that were not applicable across the different modes of treatment. Consequently, these items went on to fail the psychometric criteria, and hence were removed.

Results from this study improve our understanding of patients' experiences of relapse management. First, the process of scale development has defined four key domains for intervention and measurement (access to care, interpersonal care, information, coordination of care). This provides a clinical framework for evidence-based patient-focused relapse management. Second, the scale gives clinicians a robust mechanism for measuring the outcomes of relapse management. Of note, 75% of patients endorsed "excellent" on the single item question of quality of care, supporting the concern that simple patient satisfaction scores and global indicators of quality, may present limited and optimistic pictures [25], unlikely to be sufficient to provide the evidence base to evaluate and improve complex service delivery [26].

This study has some limitations. First, the sample sizes were relatively small, compared to other scale development studies. Second, although the interview sample included patients from two hospital sites, the postal survey and the trial sample were recruited from one hospital setting. Further psychometric evaluation of the scale is needed in a larger diverse sample of patients across a number of hospital sites. Cognitive interview techniques [27] could also be used to identify whether any other issues of importance regarding MS relapse management could be added. It is also important to evaluate the measure using other psychometric paradigms, for example Rasch analysis or item response theory [28,29]. Additional items could also be generated that could discriminate amongst those who rate the care as excellent. Rasch analysis in particular can aid the selection of these additional items. Rasch analysis can also further assess the unidimensionality of the scales. Finally, although patients' perceptions of care delivery are crucial, it is important that correlations with an objective measure of the quality of medical care are established before any public policy decisions can be made.

In conclusion, we have developed a measure of patients' experiences of treatment for MS relapses. It has robust psychometric properties, and could be extended to compare patients' experiences of relapse management across other treatments and locations (e.g. GP surgeries).

## Conclusions

The MSRMS is a reliable and valid measure of patients' experiences of MS relapse management. Results demonstrate that patients' experiences of relapse management can be measured. The MSRMS is a powerful tool for determining which services to develop, support and ultimately commission.

## Competing interests

The authors declare that they have no competing interests.

## Authors' contributions

JH conceived and designed the study. AR collected the data, analysed and interpreted the data, and drafted the manuscript. BP, JC, AJT and JH were involved in guiding the study including the design and coordination. All authors contributed to the interpretation of data and writing of the manuscript. All authors read and approved the final manuscript.

## Endnotes

1. I considered my most recent relapse to be: Mild, Moderate or Severe

2. Standard error of measurement (SEM) = SD × √(1- alpha)

3. Pearson's product-moment correlations

4. Overall, how would you rate the quality of care you received for your relapse? 1 = poor, 2 = fair, 3 = good, 4 = very good, 5 = excellent

5. Overall, how would you rate the quality of care you received for your relapse? 1 = poor, 2 = fair, 3 = good, 4 = very good, 5 = excellent

6. Independent samples t-test

## Supplementary Material

Additional file 1 **Copy of the MSRMS**. Click here for file

Additional file 2 **Scoring methods for MSRMS**. Click here for file
