# Supplementary material for: A tool to measure the attributes of receiving IV therapy in a home versus hospital setting: the Multiple Sclerosis Relapse Management Scale (MSRMS)
Source: Health Qual Life Outcomes. 2011 Sep 26;9:80. doi: 10.1186/1477-7525-9-80 (PMC3190327; doi:10.1186/1477-7525-9-80)
Supplement: Additional file 1 — Copy of the MSRMS. [file 1477-7525-9-80-S1.DOC]

**INSTRUCTIONS: Please think of your most recent relapse that was treated by steroids when filling in this questionnaire. Please answer every question by ticking the box that best describes your situation**

**A. ACCESS TO CARE**

**A1.** **How difficult was it to get hold of a health care professional (MS nurse, GP or consultant) regarding your relapse?**

🞎1 Very difficult

🞎2 Somewhat difficult

🞎3 Not really difficult

🞎4 Not at all difficult

**A2.** **How do you feel about the length of time you had to wait to have your relapse assessed by a health care professional?**

🞎1 I was assessed as soon as I thought was necessary

🞎2 I was assessed in reasonable time

🞎3 I should have been assessed a bit sooner

🞎4 I should have been assessed a lot sooner

**A3.** **Do you feel that your relapse was quickly acted upon?**

🞎1 Yes, definitely

🞎2 Yes, to some extent

🞎3 No, not especially

🞎4 No, not at all

**A4. Did you have any difficulties getting the relapse acknowledged by a health care professional?**

🞎1 Yes, definitely

🞎2 Yes, to some extent

🞎3 No, not especially

🞎4 No, not at all

**A5. Did you feel frustrated because your relapse was assessed by someone who didn’t know you?**

🞎1 Yes, definitely

🞎2 Yes, to some extent

🞎3 No, not especially

🞎4 No, not at all

**A6. Was there a time while you were waiting for intravenous steroid treatment that you were uncertain whether you will be able to have the steroids or not?**

🞎1 Very uncertain

🞎2 Somewhat uncertain

🞎3 Not really uncertain

🞎4 Not at all uncertain

**B. INFORMATION**

**B1. How much information about the effectiveness of steroids were you given before the treatment?**

🞎1 Quite a lot

🞎2 Some

🞎3 Not a lot

🞎4 None

**B2. How much information were you given on what kind of things you should and shouldn’t do?**

🞎1 Quite a lot

🞎2 Some

🞎3 Not a lot

🞎4 Not at all

**B3. How much information were you given on steroids and its effects?**

🞎1 Quite a lot

🞎2 Some

🞎3 Not a lot

🞎4 Not at all

**B4. How much information were you given on how long the relapse may last?**

🞎1 Quite a lot

🞎2 Some

🞎3 Not a lot

🞎4 Not at all

**B5. How much information were you given on the effectiveness of steroids?**

🞎1 Quite a lot

🞎2 Some

🞎3 Not a lot

🞎4 Not at all

**B6. How much information were you given on the cause of relapse?**

🞎1 Quite a lot

🞎2 Some

🞎3 Not a lot

🞎4 Not at all

**B7. Did the nurse answer any questions that you asked?**

🞎1 Answered all of my questions

🞎2 Answered some of my questions but not all

🞎3 Did not answer any of my questions

**C. INTERPERSONAL CARE**

**C1. Did you feel that you got to know the nurse who administered the steroids personally?**

🞎1 Yes, definitely

🞎2 Yes, to some extent

🞎3 No, not especially

🞎4 No, not at all

**C2. Did you feel that your family got to know the nurse who administered the steroids personally?**

🞎1 Yes, definitely

🞎2 Yes, to some extent

🞎3 No, not especially

🞎4 No, not at all

**C3. Did you feel that the nurse who administered the steroids understood the symptoms that you had?**

🞎1 Yes, definitely

🞎2 Yes, to some extent

🞎3 No, not especially

🞎4 No, not at all

**C4. Did you have a chance to have a one-to-one relationship with the nurse who administered the steroids?**

🞎1 Yes, definitely

🞎2 Yes, to some extent

🞎3 No, not especially

🞎4 No, not at all

**C5. Were you able to confide in the nurse in private about any concerns?**

🞎1 Yes, definitely

🞎2 Yes, to some extent

🞎3 No, not especially

🞎4 No, not at all

**C6. Did you feel that the nurse who administered the steroids cared about you personally?**

🞎1 Yes, definitely

🞎2 Yes, to some extent

🞎3 No, not especially

🞎4 No, not at all

**C7. Did the nurse who administered the steroids ask how good or bad you are feeling?**

🞎1 Yes, definitely

🞎2 Yes, to some extent

🞎3 No, not especially

🞎4 No, not at all

**C8. How much time did the nurse spend with you?**

🞎1 Quite a lot

🞎2 Some

🞎3 Not a lot

🞎4 Not at all

**C9. Do you feel that your needs were met?**

🞎1 Yes, definitely

🞎2 Yes, to some extent

🞎3 No, not especially

🞎4 No, not at all

**C10. Do you feel that your needs on when you wanted the steroids to be administered were met?**

🞎1 Yes, definitely

🞎2 Yes, to some extent

🞎3 No, not especially

🞎4 No, not at all

**C11. Did you feel that the nurse who administered the steroids focused their attention on you?**

🞎1 Yes, definitely

🞎2 Yes, to some extent

🞎3 No, not especially

🞎4 No, not at all

**C12. Did you feel that the nurse who administered the steroids was chatty?**

🞎1 Yes, definitely

🞎2 Yes, to some extent

🞎3 No, not especially

🞎4 No, not at all

**C13. Did you feel that the nurse who administered the steroids was focused on effective and quick treatment?**

🞎1 Yes, definitely

🞎2 Yes, to some extent

🞎3 No, not especially

🞎4 No, not at all

**C14. Did you receive support and comfort from the nurse who administered the steroids?**

🞎1 Yes, definitely

🞎2 Yes, to some extent

🞎3 No, not especially

🞎4 No, not at all

**C15. Did you feel that you were not on your own because someone was there to help you?**

🞎1 Yes, definitely

🞎2 Yes, to some extent

🞎3 No, not especially

🞎4 No, not at all

**C16. Did you feel confident that the nurse who administered the steroids can deal with any problems that arose?**

🞎1 Yes, definitely

🞎2 Yes, to some extent

🞎3 No, not especially

🞎4 No, not at all

**C17. Did you feel that the nurse who administered the steroids was interested in your thoughts and opinions?**

🞎1 Yes, definitely

🞎2 Yes, to some extent

🞎3 No, not especially

🞎4 No, not at all

**C18. Overall, how well organised was the relapse service?**

🞎1 Very well organised

🞎2 Somewhat organised

🞎3 Not really organised

🞎4 Not at all organised.

**D. COORDINATION OF CARE**

D1. How concerned were you about catching any infection while you were being treated with intravenous steroids?

🞎1 Very much

🞎2 Somewhat

🞎3 Not really

🞎4 Not at all

**D2. How convenient was the treatment?**

🞎1 Very convenient

🞎2 Somewhat convenient

🞎3 Not really convenient

🞎4 Not at all convenient

**D3. How convenient was the timing of the treatment?**

🞎1 Very convenient

🞎2 Somewhat convenient

🞎3 Not really convenient

🞎4 Not at all convenient

**D4. How comfortable was the setting in which you were treated?**

🞎1 Very comfortable

🞎2 Somewhat comfortable

🞎3 Not really comfortable

🞎4 Not at all comfortable

D5. Did you feel comfortable because you had things to do to pass the time with while you were being treated?

🞎1 Very comfortable

🞎2 Somewhat comfortable

🞎3 Not really comfortable

🞎4 Not at all comfortable

**D6. How calm and relaxed was the environment in which you were treated?**

🞎1 Very calm and relaxed

🞎2 Somewhat calm and relaxed

🞎3 Not really calm and relaxed

🞎4 Not at all calm and relaxed

**D7. Were you treated in a comfortable position?**

🞎1 Yes, definitely

🞎2 Yes, to some extent

🞎3 No, not especially

🞎4 No, not at all

**D8. How well do you think the cannula was put in?**

🞎1 Very well

🞎2 Quite well

🞎3 Not really well

🞎3 Not at all well

**D9. How practised do you feel the nurse was in infusing the steroids?**

🞎1 Very much

🞎2 Somewhat

🞎3 Not really

🞎4 Not at all

**D10. Did you feel that your blood pressure was monitored appropriately?**

🞎1 Yes, definitely

🞎2 Yes, to some extent

🞎3 No, not especially

🞎4 No, not at all

**D11. Did you feel that the speed in which the steroids were administered was appropriate for you?**

🞎1 Yes, definitely

🞎2 Yes, to some extent

🞎3 No, not especially

🞎4 No, not at all
