# Supplementary material for: A tool to measure the attributes of receiving IV therapy in a home versus hospital setting: the Multiple Sclerosis Relapse Management Scale (MSRMS)
Source: Health Qual Life Outcomes. 2011 Sep 26;9:80. doi: 10.1186/1477-7525-9-80 (PMC3190327; doi:10.1186/1477-7525-9-80)
Supplement: Additional file 2 — Scoring methods for MSRMS. [file 1477-7525-9-80-S2.DOC]

**Scoring methods for MSRMS**

The following items are reversed scored (so that low scores = better care experiences) before items in each subscales are summed: A1, A4, A5, A6, D1

Access to care = revA1 + A2 + A3 + revA4 + revA5 + revA6.

Information = B1 + B2 + B3+ B4+ B5 + B6 + B7.

Interpersonal care = C1 + C2 + C3 + C4 + C5 + C6 + C7 + C8+ C9+ C10 + C11 + C12 + C13 + C14 + C15 + C16 + C17 + C18.

Coordination of care = revD1 + D2 + D3 + D4 + D5 + D6 + D7 + D8+ D9 + D10 + D11.

All subscales scores are then transformed on a scale of 0-100 using the following equation:

100 x (observed score - minimum score)

[maximum score - minimum score]

For respondents with missing data, missing values were imputed using a respondent-specific mean score in cases where at least 50% of the items in a scale had been completed.
